# My Baby Versus the World: Fathers' Neural Processing of Own‐Infant, Unfamiliar‐Infant, and Romantic Partner Stimuli

**DOI:** 10.1002/hbm.70324

**Published:** 2025-08-12

**Authors:** Philip Newsome, Anthony G. Vaccaro, Sofia I. Cárdenas, Narcis A. Valen, Yael H. Waizman, Elizabeth C. Aviv, Gabriel A. León, Jonas T. Kaplan, Darby E. Saxbe

**Affiliations:** ^1^ Department of Psychology University of Southern California Los Angeles California USA

**Keywords:** fatherhood, infant schema, mentalizing, parenting brain

## Abstract

Parents activate brain regions linked with social cognition, reward processing, and emotion when viewing their own infant. Neural responses to own‐infant stimuli may be driven by familiarity, self‐relevance, or by the unique features of infant faces. The current study sought to clarify these distinctions in first‐time fathers by contrasting video stimuli of their infant, an unfamiliar infant, and their pregnant partner. In addition, we examined associations with fathers' self‐reported bonding and parenting stress. Fathers (*n* = 32) scanned approximately 8 months after the birth of their first child completed an fMRI scan while watching videos of their infant, an unfamiliar infant, their pregnant partner, and an unfamiliar pregnant woman. We compared neural responses to the participant's own infant to these other stimuli using both traditional univariate methods and multivariate searchlight analyses. Lastly, we ran additional multivariate pattern analyses to determine discriminability between infant and adult stimuli, as well as familiar and unfamiliar stimuli. Consistent with previous studies, fathers showed greater activation to their own infant versus an unfamiliar infant in regions including the precuneus, posterior cingulate, orbitofrontal cortex, and inferior frontal gyrus. In addition, fathers exhibited heightened activation to their own infant versus their partner in the precuneus. Fathers who reported stronger antenatal and postpartum bonding and lower parenting stress 3 months after birth subsequently showed stronger activation in the precuneus and posterior cingulate to their own infant. Multivariate pattern analyses revealed that in addition to these regions, the parahippocampus differentiated own‐infant stimuli versus other conditions. Neural patterns distinguished infant/adult and familiar/unfamiliar stimuli in mentalizing, visual, and affective areas. These findings replicate and extend previous research on the parental brain and suggest that cortical midline mentalizing network regions, as well as visual and reward areas, are particularly important in first‐time fathers' processing of their own infants.

## Graphical abstract

First‐time fathers processed own‐infant stimuli in mentalizing, emotion regulation, and reward processing regions, and posterior midline responses tracked with bonding and parental stress. Whole‐brain searchlights highlighted similar regions as our univariate contrasts, along with the parahippocampus and visual regions. Exploratory MVPA revealed unique contributions of familiarity and infancy dimensions.

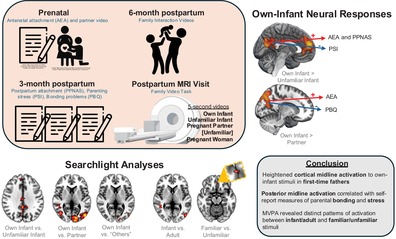

**Summary**

- Univariate fMRI analyses found that first‐time fathers showed heightened activation to their own infant, compared to an unfamiliar infant and their partner, in regions subserving mentalizing, emotion regulation, and reward processing.
- MVPA revealed distinct responses to own‐infant stimuli, compared to both self‐relevant and infant stimuli.
- Father's parenting stress and bonding with their infants were associated with stronger own‐infant responses in the posterior midline of the cortex.

## 1 Introduction

Human fathers often make important contributions to infant care, and there is emerging evidence that their neurobiology may reflect their participation in parenting (Feldman et al. 2019). While most human parenting fMRI research has focused on how mothers respond to images of their infants, there is a growing literature showing that fathers also show distinct responses to their own infants (Provenzi et al. 2021). However, past studies suffer from a potential confound: own‐infant tasks conflate two dimensions of salience, infant features and familiarity. Thus, the current study leverages univariate and multivariate fMRI approaches and introduces a new condition, images of one's own partner, to better understand the specificity of fathers' responses to their infant.

Infant faces, characterized by features such as large eyes, a round head, and a small chin—collectively known as “baby schema” or *kindchenschema*—evoke strong affective, hormonal, and neural responses, motivating approach behaviors and caregiving in adults (Hahn and Perrett 2014; Glocker et al. 2009; Lorenz 1981). Mothers have stronger responses to infant faces compared to non‐mothers, particularly in regions involved in emotion regulation (e.g., insula, amygdala, prefrontal cortex), reward processing (e.g., orbitofrontal cortex), and social cognition (e.g., precuneus; Plank et al. 2022; Bjertrup et al. 2021; Zhang et al. 2020). Mothers display an additional preference for their own infant's face compared to unfamiliar infants. Early work from Ranote et al. (2004) found stronger activation in the bilateral orbitofrontal cortex (OFC) and medial prefrontal cortex (mPFC) to own‐infant video stimuli compared to videos of an unfamiliar infant. Using static images, Leibenluft et al. (2004) found activation in the posterior cingulate cortex (PCC), precuneus, inferior frontal gyrus (IFG), superior temporal sulcus (STS), fusiform gyrus, anterior cingulate cortex (ACC), the amygdala, and the insula to be stronger when mothers viewed their own infant versus an unfamiliar infant. In a more recent study, Hoekzema et al. (2017) show that activation in the IFG and PCC to mothers' own infant, versus an unfamiliar infant, tracks with structural changes across the transition to parenthood, further suggesting these regions adapt to support the new caregiving role.

Neural processing of own‐infant stimuli has received less study in fathers. Notably, fathers do not physically undergo pregnancy and may exhibit overlapping, but distinct, neuroendocrinological responses to their offspring that support complementary caregiving (Açıl et al. 2025). In a sample of 30 families, Atzil et al. (2012) found that mothers' responses in limbic regions, including the amygdala, nucleus accumbens, and ACC, while viewing videos of their own infant were stronger than fathers' responses. On the other hand, fathers showed greater activation in social cognition regions, including the dorsal prefrontal cortex, precuneus, and superior temporal gyrus (STG). Similarly, Abraham et al. (2014) found that when viewing their own infant, primary caregiver mothers exhibited increased amygdala activation; secondary caregiver fathers activated the superior temporal sulcus (STS), and primary caregiving fathers showed both heightened amygdala and STS activation. Kuo et al. (2012) found own‐infant pictures to evoke stronger activation in fathers, particularly cortical regions subserving mentalizing processes (IFG, middle temporal gyrus, and supramarginal gyrus) when compared to neural responses to an unfamiliar infant. More recent work in first‐time fathers observed stronger activation to their own infant compared to an unfamiliar infant in the precuneus, PCC, IFG, inferior parietal gyrus, angular/precentral gyrus, inferior/posterior temporal gyrus, and middle and superior occipital lobes, regions known to be important for mentalizing and empathy (Paternina‐Die et al. 2020). In sum, engaging with an infant, particularly one's own child, evokes specialized brain responses in regions involved in mentalizing processes, along with reward processes and emotion areas of the brain. Mentalizing—the ability to interpret others' internal states—may support sensitive parenting by facilitating empathy for one's child. In addition, mentalizing may be uniquely important within the context of the parent–infant attachment relationship because young children are unable to clearly communicate their needs (Borelli et al. 2020; Shai and Belsky 2011). While these functions seem distributed across cortical and subcortical regions in mothers, the emerging literature on the fathering brain suggests predominantly cortical responses to their own baby.

New parents' neural responses to their own infants may be driven by multiple overlapping features of own‐infant stimuli, including the schematic features of babies, their familiarity and self‐relevance, or by the unique caregiving salience of one's own child. The current study seeks to tease apart these different features to better establish what is unique about neural responses to own‐child stimuli. Social neuroscience research has theorized that self‐relevant stimuli, such as images of one's own face, are processed in regions including the anterior mPFC and ACC, as well as posterior midline regions such as the precuneus and PCC (Levorsen et al. 2023; Lieberman et al. 2019). There appears to be some overlap in terms of how the brain responds to self‐stimuli and personally familiar stimuli, with evidence that personally familiar faces elicit neural activation that is distinct from novel faces (e.g., unfamiliar strangers). A meta‐analysis found that familiar others, such as family members, may elicit more posterior mentalizing network activation (PCC, temporoparietal junction) whereas self‐stimuli elicit more anterior activation (ACC, anterior insula; Qin et al. 2012). A 2024 meta‐analysis of 79 fMRI studies focused on neural responses to affiliative figures, including one's own babies, family members, romantic partners, and friends (Bortolini et al. 2024). It included 17 studies that used own‐infant visual stimuli (faces or video), typically contrasted with unfamiliar infants, and another 14 studies that used face or video images of one's romantic partner, contrasted with comparison conditions featuring adults (strangers or friends). Both sets of studies unearthed similar findings, with clusters appearing in regions related to reward and motivation (ventral pallidum, thalamus, striatum, and vmPFC); social bonding (medial preoptic area, septo‐hypothalamic region); social cognition and embodied simulation (extended amygdala, PCC and precuneus, anterior insula, inferior parietal lobule, and IFG); and salience‐related regions (amygdala, ACC, and insula). However, most of the own‐infant studies focused on mothers, and no studies contrasted one's own infant with their own partner. This is a gap in the literature because contrasting one's infant from one's partner can help disentangle the effects of familiar, self‐relevant affiliative figures from the specific salience of one's own baby.

Several studies have attempted to relate neural processing of own‐infant stimuli to individual differences in parenting. Barrett et al. (2012) found that mothers who reported stronger parent–infant bonding, or emotional connection towards their child, and less parental stress exhibited stronger amygdala activation to viewing their own infant versus an unfamiliar infant. Other work in mothers has shown parenting stress to be inversely related to activation in the OFC, a cortical reward‐processing region (Noriuchi et al. 2019). There are fewer studies on how fathers' neural processing relates to their parenting experience. In the aforementioned Abraham et al. (2014), behaviorally coded parent–child synchrony was associated with STS activation in fathers. Kuo et al. (2012) reported an inverse relationship between parental sensitivity and reciprocity, two behaviorally coded measures of attunement, and activation in OFC for their own‐infant > unfamiliar‐infant contrast in a small sample of fathers. More work is needed to understand how brain function relates to parenting, particularly in first‐time fathers.

While the majority of studies on parents' neural responses to their own babies have relied on univariate contrasts, multivariate pattern analysis (MVPA) may reveal additional features of own‐infant neural processing. Unlike univariate contrasts, which rely on mean differences in activation, MVPA can capture differences in regional neural patterns between stimuli (Haxby et al. 2014). Neural patterns, rather than overall activation, may be particularly relevant for understanding potential differences in how similar stimuli are processed within the same region, especially when the same region is implicated in processing both categories of stimuli (Brooks et al. 2021; Jimura and Poldrack 2012). Two previous studies by Diaz‐Rojas and colleagues have used MVPA to investigate neural processing of parenting‐related stimuli across the transition to fatherhood. The first found that the patterns in the left insula while viewing parent–infant interaction videos from a first‐person point of view could significantly distinguish between expectant fathers and childless men, despite the two groups not showing any significant differences in mean activation throughout the brain (Diaz‐Rojas et al. 2021). Additionally, in both expectant fathers and non‐fathers, patterns of activation in the temporal poles predicted if participants had any recent caregiving experience; importantly, this relationship was not observed when considering the univariate brain maps. In the second study, activation patterns in the dorsomedial PFC when viewing infant stimuli could significantly distinguish between expectant fathers and control group men during their partners' mid and late pregnancy, but not early pregnancy (Diaz‐Rojas et al. 2023).

These findings show that MVPA is sensitive to aspects of neural processing not necessarily revealed by univariate contrasts, and that these processes may directly relate to sociocognitive adaptation that occurs in the transition to new fatherhood. With this in mind, MVPA may be beneficial to understanding nuances in the neural processes involved when new fathers view their own infants, and how these processes relate to their parenting experience. MVPA methods may also help us understand the contributions of constructs such as personal familiarity in the processing of one's own infant. Visconti di Oleggio Castello et al. (2017) trained classifiers on a set of personally familiar (including own) and unfamiliar faces and found significant familiarity classification across identities in bilateral middle temporal, right anterior fusiform, right IFG, temporoparietal junction (TPJ), PCC, and mPFC areas.

Thus, the current study aimed to employ univariate analyses and MVPA to further understand unique processing of one's own child in new fathers, as well as explore the roles of general infant‐feature processing and familial relevance. We examined first‐time fathers' neural responses to videos of their own infant, unfamiliar infant, and their own pregnant partner and related these brain processes to antenatal and postpartum bonding, postpartum stress, and baby age. Further, we employed MVPA to determine if we can distinguish between task stimuli based on patterns of activation across the brain. Taken together, the current study has three main hypotheses.

Hypothesis 1 *We tested univariate contrasts between own‐infant, unfamiliar‐infant, and partner stimuli. (1a) Consistent with previous literature, we hypothesized that fathers would show greater activation in cortical regions implicated in mentalizing (TPJ, STS, PCC, and precuneus) and emotion regulation (e.g., dlPFC, IFG, OFC) when viewing videos of their own infant versus an unfamiliar infant. (1b) We also contrasted the own‐infant condition with a romantic partner condition. As this was the first study to compare fathers' responses to infant and partner stimuli, we did not have specific predictions*.

Hypothesis 2 *Based on prior work on how fathers' neural responses relate to parenting experience and behaviors, we hypothesized that fathers who showed greater activation in the OFC and STS to their own infant versus an unfamiliar infant would report better adjustment to parenthood, operationalized as greater antenatal and postpartum bonding, lower parenting stress, and fewer parent–child bonding problems. We expected to find similar associations with activation in posterior midline regions subserving mentalizing processes (e.g., PCC and precuneus), since greater engagement in social cognition regions may underlie better adjustment to parenthood*.

*Exploratory Aim 1:* We tested whether brain activation for the own‐infant > partner contrast was associated with adjustment to parenthood. We tested whether duration of parental experience (infant age and primary caregiver time) was associated with activation to either own‐infant > unfamiliar‐infant or own‐infant > partner contrasts.

Hypothesis 3 *We explored similarities between the univariate and MVPA results; specifically, whether discriminability between own‐infant and unfamiliar‐infant videos emerged in similar regions to the univariate analysis or in different or additional regions*.

*Exploratory Aim 2:* Using MVPA, we examined whether discriminability was driven more by infancy (infant vs. adult partner) or familiarity (familiar vs. unfamiliar) and which regions contributed most to the decision function.

## 2 Materials and Methods

### 2.1 Participants

Data for the current study were drawn from a longitudinal study on the transition to parenthood conducted in the western United States. The study recruited 100 different‐sex cohabitating couples from the greater Los Angeles area through fliers posted in obstetricians' offices, at community health clinics, and on social media. All parents were expecting their first baby. Families began the study during the female partner's second trimester of pregnancy and were followed over the first year postpartum. A total of 42 fathers opted to participate in the MRI portion of the study, which started in the prenatal period. Of these, 38 fathers returned to complete a postpartum scan. Two subjects were not administered the fMRI task properly, and family video task stimuli were unavailable for four subjects who were unable to come into the lab because of the COVID‐19 pandemic. Thus, the current study includes 32 fathers who had video available from both their partner and infant and who completed the postpartum MRI visit. Demographics for the 32 fathers who completed the postpartum fMRI visit are shown in Table 1.

**TABLE 1:** Demographics and key study variables.

Characteristic	*N* = 32
Parent's age at prenatal visit in years, mean (SD)	31.66 (4.25)
Gestational age at the prenatal visit in weeks, mean (SD)	29.02 (4.72)
Gestational age at birth in weeks (SD)	39.89 (1.05)
Infant weight at birth in pounds (SD)	6.83 (0.80)
Infant sex assigned at birth, *N* (%)	19F (59.4)
Preterm births, *N* (%)	2 (0.06)
Parent's age at postpartum fMRI visit in years, mean (SD)	32.90 (4.31)
Infant age at postpartum fMRI scan in months, mean (SD)	8.01 (3.32)
Parent's time spent as primary caregiver in hours/week, mean (SD)	18.54 (12.01)
Race/ethnicity, *N* (%)	
White	10 (31%)
Black or African American	3 (9.4%)
Hispanic or Latino/a	10 (31%)
American Indian or Alaska Native	0 (0%)
Asian American and Pacific Islander	7 (22%)
Other	2 (6.3%)
Highest education level, *N* (%)	
High school graduate/GED	1 (3.1%)
Some college	5 (16%)
Associate's degree	1 (3.1%)
Bachelor's degree	11 (34%)
Master's degree	7 (22%)
Professional or doctoral degree	7 (22%)
Household income, *N* (%)	
Less than 25k	0 (0%)
25k–50k	3 (10%)
50k–75k	6 (21%)
75k–100k	7 (24%)
100k–125k	1 (3.4%)
More than 125k	12 (41%)
Unknown	3 (9.4%)

Abbreviations: F = female, SD = standard deviation.

### 2.2 Procedure

The first laboratory visit occurred during mid‐to‐late pregnancy. During that visit, couples took part in discussion tasks that were videotaped. Fathers also filled out questionnaires including the antenatal bonding questionnaire. At 3 months postpartum, couples completed remote questionnaires that included the postpartum bonding, bonding problems, and parenting stress questionnaires. One subject did not complete the three‐month postpartum questionnaires; thus, analyses with those data include only 31 subjects. The postpartum in‐person visit occurred approximately 6 months after birth. In this visit, infants were seated in a highchair and did a brief temperament assessment task adapted from the Laboratory Temperament Assessment Battery (Lab‐TAB). Fathers returned for a neuroimaging scan on average 2 weeks (46.31 days) after their 6‐month lab visit. The postpartum MRI visit included the Family Video Task, which is summarized in the following section.

**FIGURE 1:**
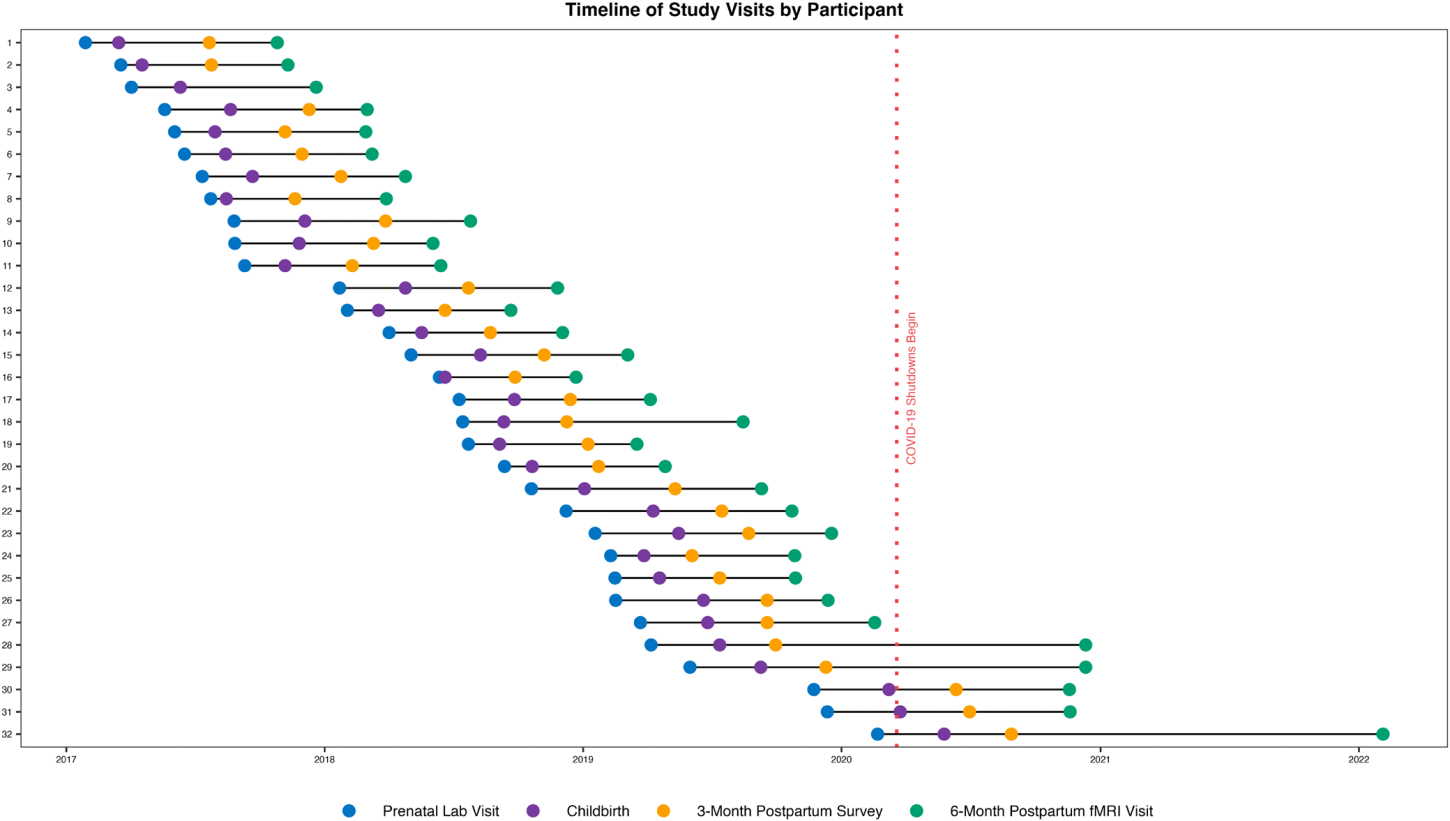
Study timeline for each subject (*n* = 32). Each horizontal line represents a subject from the sample with usable fMRI data from the task.

The COVID‐19 pandemic occurred after 27 fathers had already completed their postpartum visit and scan. Postpartum MRI scans for these 27 fathers were completed when parents' infants were on average 6.95 months (range = 5.79–9.73 months). However, as shown in Figure 1, five subjects' visits were delayed due to the closure of the neuroimaging center during the COVID‐19 pandemic and did not occur until an average of 13.75 months after birth (range = 7.89–20.38 months). Across the whole sample, the postpartum MRI scan occurred on average 8.01 months postpartum (range = 5.79–20.38 months). Independent‐samples *t*‐tests confirmed measures of interest for our study did not differ between fathers who participated pre‐pandemic versus during the pandemic (all *p*‐values > 0.25).

### 2.3 Measures

#### 2.3.1 Family Video Task (FVT)

At the postpartum scan, fathers viewed five‐second silent video clips depicting their partner (recorded during the prenatal visit conflict discussion), an unfamiliar pregnant woman (recorded in the same lab setting), their own infant (recorded during the Lab‐TAB), and an unfamiliar infant (also recorded during the Lab‐TAB). While fathers were present during the filming of their partner, they were not present for the filming of their infant. In addition, unfamiliar conditions (unfamiliar woman and unfamiliar infant) were standardized across participants to minimize confounding effects due to differences in stimulus content. All clips showed the face and upper body of the featured person. The clips featured a range of emotional expressions, and research assistants rated the emotional valence of the clips so that an equal number of positive and negative clips would be shown to each participant. Videos captured for the task were naturalistic, in that we did not attempt to evoke discrete emotional expressions. In the scanner, fathers were asked to use the button box to rate the emotional valence of each clip from −2 (most negative) to +2 (most positive); this ensured they were paying attention to the clips throughout the task. Each task run included five 12‐s trials per condition, with condition order optimized using a genetic algorithm (Wager and Nichols 2003) to balance hemodynamic responses—each condition was shown 10 total times per run. The FVT follows the structure of similar tasks contrasting self‐relevant and unfamiliar social stimuli (Saxbe et al. 2016; Saxbe, Del Piero, Immordino‐Yang, et al. 2015; Saxbe, Del Piero, and Margolin 2015).

#### 2.3.2 Antenatal Bonding (AEA)

Fathers' antenatal (i.e., prenatal) bond with their unborn child was measured using the *Paternal Antenatal Attachment Scale* (PAAS). The PAAS is a 16‐item questionnaire designed to measure fathers' self‐reported thoughts, feelings, attitudes, and behaviors pertaining to their developing baby *in the last 2 weeks* (Condon 1993). Each item was measured using a 5‐point Likert‐type scale. For example, fathers were asked *over the past 2 weeks, when I think about the developing baby, I get feelings which are*—and responses for this item ranged from “1” = *very sad* to “5” = *very happy*. Higher scores indicate a stronger sense of bonding with their soon‐to‐be born infant. The PAAS has been found to have adequate psychometric properties, and the internal reliability in our sample was also adequate (Condon 1993; *α* = 0.87, *ω* = 0.92).

#### 2.3.3 Postpartum Bonding (PPNAS)

Postpartum father‐infant bonding was measured using the *Paternal Postnatal Attachment Scale (PPNAS)*, a 19‐item questionnaire that measures fathers' self‐reported thoughts, feelings, attitudes, and behaviors about or in relation to their newborn (Condon et al. 2008). This scale consists of one dichotomous item, two 3‐point scale questions, seven 4‐point scale questions, and nine 5‐point scale questions. Higher scores indicate stronger postpartum bonding with their infant. The PPNAS has been found to have adequate construct validity and reliability (Condon et al. 2008); internal reliability was adequate in our sample (*α* = 0.88, *ω* = 0.91).

#### 2.3.4 Bonding Problems (PBQ)

Father‐infant bonding problems were measured using the *Postpartum Bonding Questionnaire (PBQ)*, a 25‐item questionnaire designed to detect abnormalities and problems in the parent–infant relationship and to identify those at high risk of child abuse (Brockington et al. 2006). Items are measured on a 6‐point Likert‐type scale with responses ranging from “1” = *never* to “6” = *always*. Higher scores indicate more reported problems in the father–infant relationship. This measure has been found to have adequate construct validity and was found to be internally reliable in our sample (Brockington et al. 2006; *α* = 0.88, *ω* = 0.92). One outlier was detected in these data (|*z*| > 3), resulting in 30 total subjects for our analyses using the PBQ.

#### 2.3.5 Parenting Stress (PSI)

Fathers' parenting stress at 3 months postpartum was measured using the *Parenting Stress Index—Short Form (PSI)*, a 36‐item questionnaire comprising three 12‐item subscales that assess stress in the parent–child relationship (Abidin 1995). Fathers rate their agreement with each statement using a 5‐point Likert scale (“1” = *strongly agree;* 5 = *strongly disagree*). Fathers were provided with the prompt: *Read each statement carefully, and then for each statement, select the option that best represents your level of agreement or disagreement*. Example items include: *I often have the feeling that I cannot handle things very well, I find myself giving up more of my life to meet my child's needs than I ever expected*, and *I feel trapped by my responsibilities as a parent*. Total scores are computed by summing scores from the three subscales to get a Total Stress score. Higher scores indicate more stress during the parenting experience. The PSI has demonstrated internal consistency, convergent validity, and predictive validity in ethnically diverse samples of families with infants (Barroso et al. 2016; Haskett et al. 2006). The PSI had strong reliability in our sample of fathers (*α* = 0.94, *ω* = 0.96).

#### 2.3.6 Primary Caregiver Time

At 3 months postpartum, fathers reported the number of hours they typically spent with their infant on weekdays and weekend days, using a 6‐point scale (“1” = *more than 8 h*, “6” = *less than 1 h*). Fathers also indicated the proportion of this time during which they were either alone with the infant or served as the primary caregiver using a 5‐point scale (“1” = *at least 75% of the time*, “5” = *less than 25% of the time*). These responses were used to estimate total weekly hours spent as the infant's primary caregiver by multiplying the total weekly time with the infant by the percentage of that time spent as the primary caregiver.

### 2.4 Image Acquisition

All fMRI images were collected using a Siemens 3 T MAGNETON Prisma System scanner at the University of Southern California's Dornslife Cognitive Neuroimaging Center using a 20‐channel head coil. Anatomical images were captured using a T1‐weighted magnetization‐prepared rapid gradient‐echo sequence (TR = 2300 ms, voxel size 1‐mm isotropic voxels, flip angle 9°). Functional images were acquired using a T2*‐weighted gradient‐echo, echo‐planar image sequence (TR = 2000 ms, flip angle: 90°, 90 volumes, slice thickness 2.5 mm). In line with the neuroimaging center's policy, a T2‐weighted volume was acquired for blind review by an independent radiologist. Infant and partner videos were projected on the rear screen using an LCD projector, and sound was played through the Siemens V14 sound headphone system.

### 2.5 Data Analysis

#### 2.5.1 Preprocessing

Preprocessing and initial univariate analyses were performed using FSL (www.fmrib.ox.ac.uk/fsl). Images were skull‐stripped using FSL's Brain Extraction Tool (Smith 2002). Registration to high‐resolution structural and standard space images was performed using FLIRT (Jenkinson et al. 2002; Jenkinson and Smith 2001), and registration from high‐resolution structural to standard space was further refined using FNIRT nonlinear registration (Andersson et al. 2007). We additionally performed motion correction with MCFLIRT (Jenkinson et al. 2002), slice‐timing correction using Fourier‐space time‐series phase‐shifting, spatial smoothing using a 6 mm Gaussian kernel, and grand‐mean intensity normalization of the entire 4D dataset by a single multiplicative factor.

#### 2.5.2 Univariate Analyses

Analyses were performed using FSL's FEAT tool version 6.0. We used a general linear model approach with a mixed effects design. Each component of the task was included as a regressor (own‐infant, own‐partner, unfamiliar‐infant, unfamiliar‐partner) and modeled by convolution with a double‐gamma hemodynamic response. The task period of the video was defined from the beginning to the offset of the five‐second video clips. Each of the subjects' two runs was combined in second‐level fixed‐effects analyses. Contrasts of interest were constructed as linear combinations contrasting explanatory variables (e.g., own‐infant > unfamiliar‐infant), as well as explanatory variables contrasted with rest to be used for MVPA (e.g., own‐infant > rest).

Higher‐level analyses were carried out using FMRIB's Local Analysis of Mixed Effects (FLAME; Woolrich et al. 2004). We computed group‐level activation maps for each contrast of interest across all subjects using FLAME. Multiple comparisons were accounted for by applying cluster thresholding using Gaussian Random Field theory (*z* > 3.1; *p* < 0.05) to provide a more sensitive correction than voxel‐based methods. Since some subjects' study timeline was disrupted due to the COVID‐19 pandemic, we ran additional tests controlling for infant age at the postpartum scan. We ran two final tests controlling for participants' age and years of education.

#### 2.5.3 Nonparametric Permutation Inference

We performed nonparametric permutation testing (Winkler et al. 2014) to assess associations between activation and self‐report scores. To constrain analyses to task‐relevant regions, we applied binarized, thresholded statistical maps from our univariate analyses as masks. Using FSL's Randomise tool, we generated 5000 permutations to create a null distribution and used this distribution to generate a 1‐*p‐*value brain map representing associations between brain activation and self‐report scores. Family‐wise error (FWE) was corrected for using FSL's threshold‐free cluster enhancement (TFCE) algorithm. Following this, we used PALM (Permutation of Linear Models, Winkler et al. 2014) to compute *r*‐statistic brain maps representing the strength and direction of these associations. Since FSL Randomise generated corrected 1‐*p* maps, we ran PALM with a single permutation to avoid redundancy.

#### 2.5.4 Own‐Infant Searchlight Analyses

MVPA was performed using PyMVPA (Hanke et al. 2009). First, we used a searchlight approach to classify own‐infant versus partner, own‐infant versus unfamiliar‐infant, and own‐infant versus the other two categories combined into one unfamiliar‐infant + partner condition. We opted not to include the unfamiliar‐partner condition to focus on stimuli that shared at least one feature with own‐infant stimuli (infancy or familiarity). The input to the classifier was a 4D file including *z*‐stat maps for each of the conditions versus rest, from each functional run, from each participant. This resulted in 128 concatenated images for the first two analyses (2 *z*‐stat conditions × 2 runs × 32 participants) and 192 concatenated images for the own‐infant versus unfamiliar‐infant + partner analysis (3 *z*‐stat conditions × 2 runs × 32 participants). For every voxel in the brain, a 5‐voxel radius sphere centered on that voxel was used to train and test a support vector machine (SVM). We used leave‐one‐out (LOO) cross‐validation, where one subject's data was left out in each iteration, and a model trained on the other 31 subjects was then tested on the data of the left‐out subject. This approach has been shown to produce unbiased estimation of predictive accuracy and is recommended when working with more modest samples (Vabalas et al. 2019). For the own‐infant versus unfamiliar‐infant + partner searchlight, we additionally incorporated a balancer to assure that an equal number from each condition were included in each validation fold. The resulting average accuracy was mapped to the center voxel of the sphere. We used the default setting of PyMVPA to automatically choose the SVM regularization parameter based on the norm of the data. To threshold the searchlight analyses, we used a resel‐wise Bonferroni correction based on the number of independent 5‐voxel radius spheres that could fit in the brain (~725) and divided 0.05 by this number (Kaplan and Meyer 2012; Vaccaro et al. 2022). This led to a corrected alpha of 6.89 × 10^−5^. We applied this threshold to the binomial distribution to calculate a critical value threshold of ~66.4% accuracy for significance. Given that this correction method produces a conservative significance threshold for each image, and that data between adjacent searchlight spheres may overlap and not be independent, we did not further correct for conducting multiple searchlight analyses.

#### 2.5.5 Exploratory MVPA to Classify Infant and Familiar Stimuli

We ran two sets of exploratory analyses with MVPA methods to map where, and how well overall, the neural data could discriminate between stimuli that differed either in terms of being a baby or adult (own‐infant and unfamiliar‐infant vs. own‐partner and unfamiliar‐partner), or in terms of familiarity (own‐infant and own‐partner vs. unfamiliar‐infant and unfamiliar‐partner). In the first set of analyses, we trained a general SVM classifier which used the top 10% of the most informative voxels throughout the brain from the training data and a LOO cross‐validation approach. This analysis led to one accuracy value for each characteristic. Significance was determined by running 1000 permutations to simulate classification values that might occur in the top 10% of voxels contributing to the decision function. In the second set of exploratory analyses, we used the same searchlight approach as in our previous analyses to create whole‐brain maps of discriminability based on these characteristics.

## 3 Results

### 3.1 Hypothesis 1: Contrasts With Own‐Infant Stimuli

**FIGURE 2:**
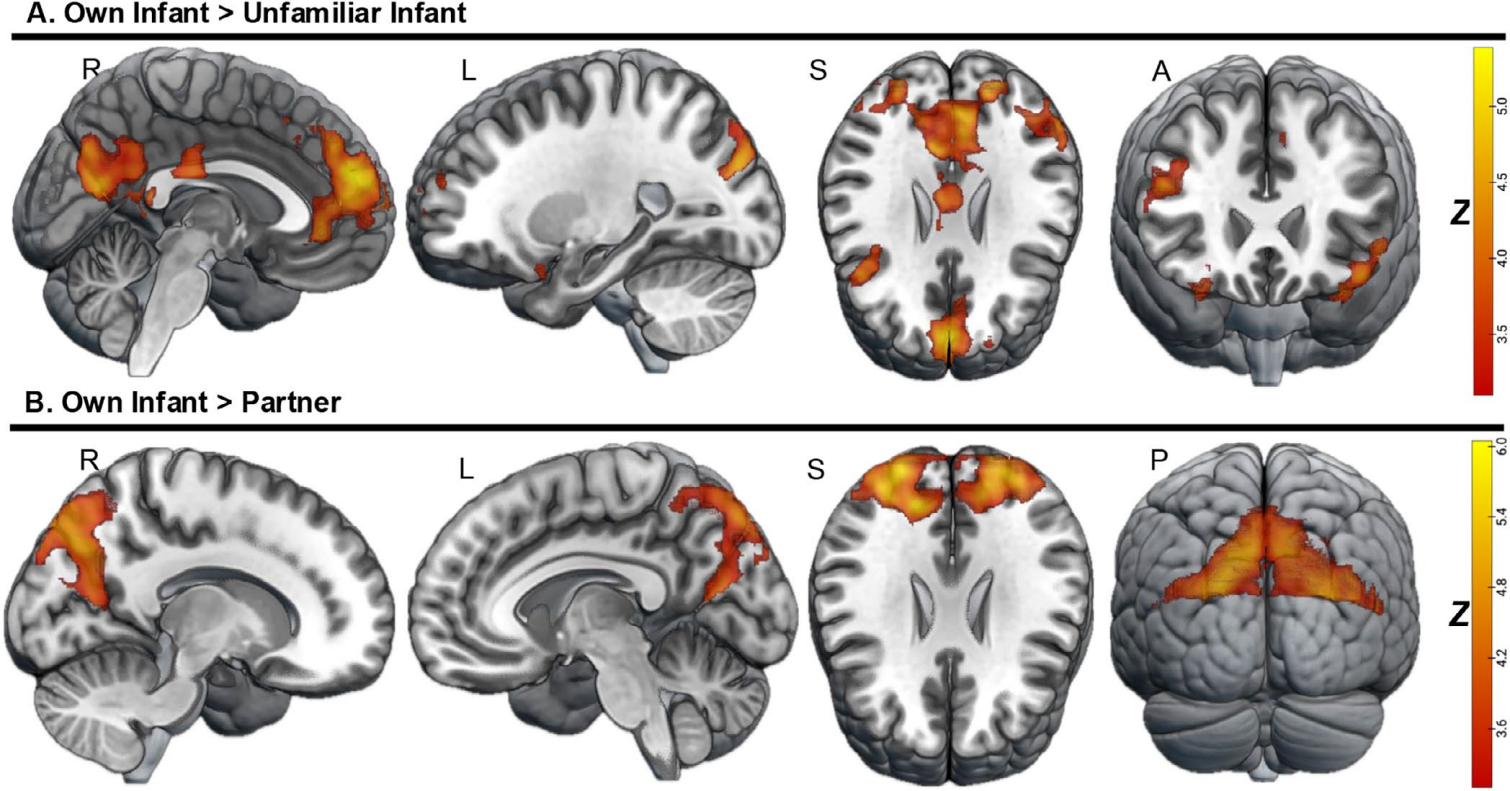
Whole‐brain BOLD activation to viewing videos of participants' own infants contrasted with an unfamiliar infant and their pregnant partner. (A) Greater BOLD activation was observed in the left precuneus (extending to the left cuneus and PCC), dorsomedial PFC (extending to the anterior PFC), left angular gyrus, right IFG (extending to the right dlPFC), bilateral OFC, and left temporal pole (extending into the left middle temporal gyrus and insula) for the own‐infant > unfamiliar‐infant contrast. (B) A cluster spanning the precuneus, cuneus, PCC, and middle occipital gyrus showed heightened activation for the own‐infant > partner contrast. Thresholding was set at *z* = 3.1.

When we tested the univariate contrasts of own‐infant > unfamiliar‐infant stimuli, we observed heightened activation in many of the regions that have been implicated in previous studies such as the precuneus and PCC, dorsomedial and anterior PFC, left angular gyrus, right IFG, bilateral OFC, and left temporal pole (Figure 2A).

When the own‐infant condition was contrasted with the own‐partner condition (Hypothesis 1b), a large cluster peaking in the precuneus and extending into the cuneus, PCC, and middle occipital gyrus showed significantly greater activation (Figure 2B). Controlling for father's age, their infant's age, or father's years of education did not affect any of the findings; thus, we chose to leave these analyses out of the manuscript for parsimony. Cluster information including size, MNI coordinates, and *z*‐scores is reported in the supplement (Table S1).

### 3.2 Hypothesis 2: Associations With Parent Bonding, Stress, and Parental Experience

**FIGURE 3:**
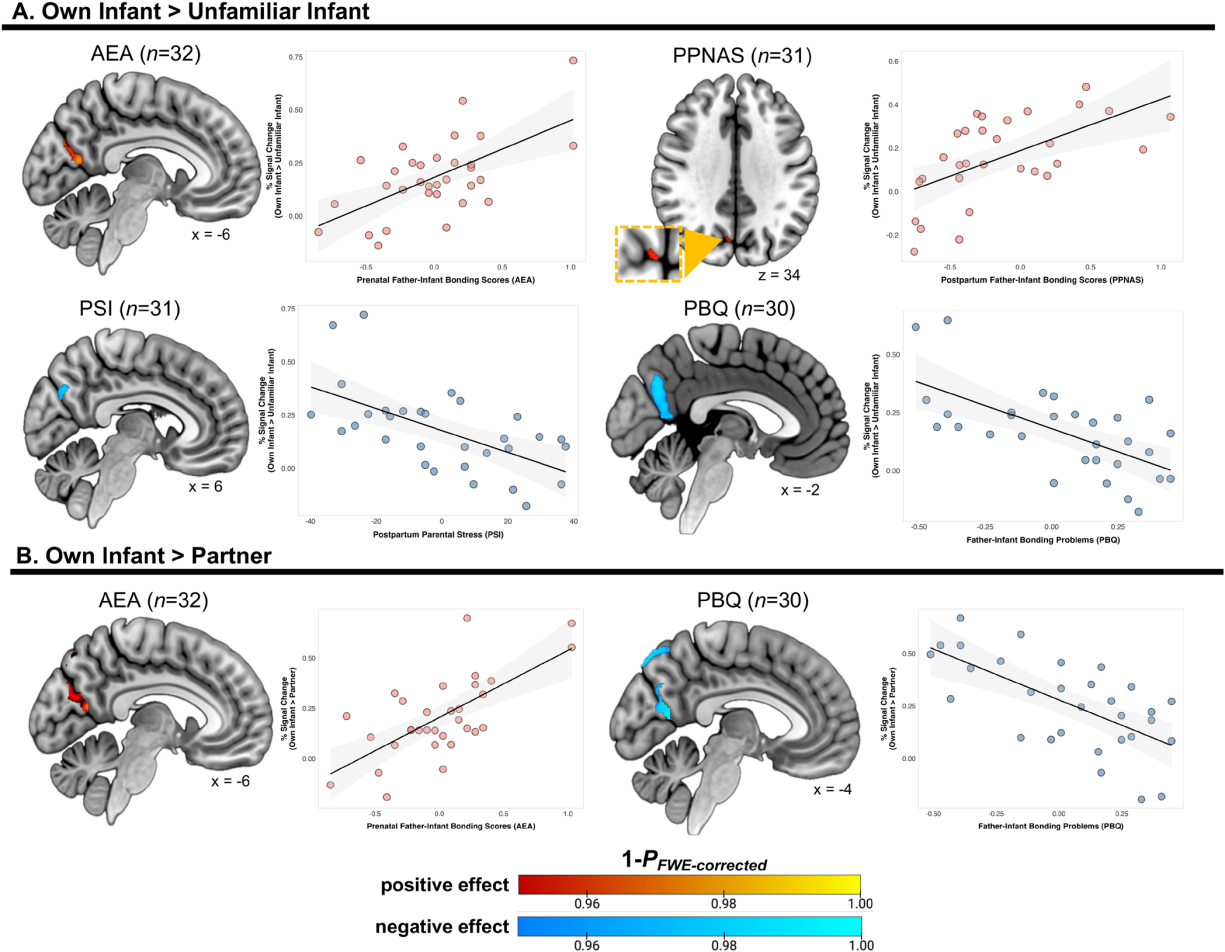
Associations between brain activation and self‐reported parenting measures (*p*_FWE_ < 0.05, TFCE‐corrected). (A) For the own‐infant > unfamiliar‐infant contrast, activation in the precuneus/PCC was positively associated with self‐reported prenatal bonding with their child, and activation in the precuneus was positively associated with father‐infant bonding 3 months postpartum. Precuneus/PCC activation was also inversely associated with parenting stress and bonding problems at 3 months postpartum. (B) For the own‐infant > partner contrast, activation in the precuneus/PCC was positively associated with prenatal bonding and negatively associated with father‐infant bonding problems at 3 months postpartum. Scatterplots plot our individual difference measures against neural activation in clusters identified by our formal nonparametric permutation inference analysis. Importantly, these plots do not represent additional ROI‐based analyses and are for illustrative purposes only.

We found that activation for the own‐infant > unfamiliar‐infant contrast in a cluster containing the bilateral PCC and precuneus was positively associated with self‐reported antenatal bonding scores (*r*_MAX_ = 0.66, *p* = 0.01; Figure 3A). In addition, father‐infant bonding at 3 months postpartum was positively associated with activation in the precuneus (*r*_MAX_ = 0.57, *p* = 0.04; Figure 3A). Parenting stress and father‐infant bonding problems at 3 months postpartum were inversely associated with activation in a single cluster spanning the precuneus and PCC (*r*_MAX_ = −0.59, *p* = 0.02 and *r*_MAX_ = −0.64, *p* = 0.01, respectively; Figure 3A). For the own‐infant > partner contrast, activation in a cluster extending across the PCC to the precuneus to the cuneus (*r*_MAX_ = 0.59, *p* = 0.02), along with three other smaller precuneus clusters (*r*_MAX_ = 0.56, *p* = 0.04, *r*_MAX_ = 0.51, *p* = 0.05, and *r*_MAX_ = 0.56, *p* = 0.04, respectively) was positively associated with antenatal bonding. Lastly, the bilateral precuneus was negatively associated with father‐infant bonding problems (*r*_MAX_ = −0.73, *p* < 0.01; Figure 3B).

No associations between brain activation and infant age at the scan or primary caregiver time survived our cluster corrected significance threshold for either contrast (all *p*s > 0.05). Information on cluster size, MNI coordinates, 1‐*p* values, *r*‐statistics is in the Supplement (Table S2).

### 3.3 Hypothesis 3: Own‐Infant Searchlights

**FIGURE 4:**
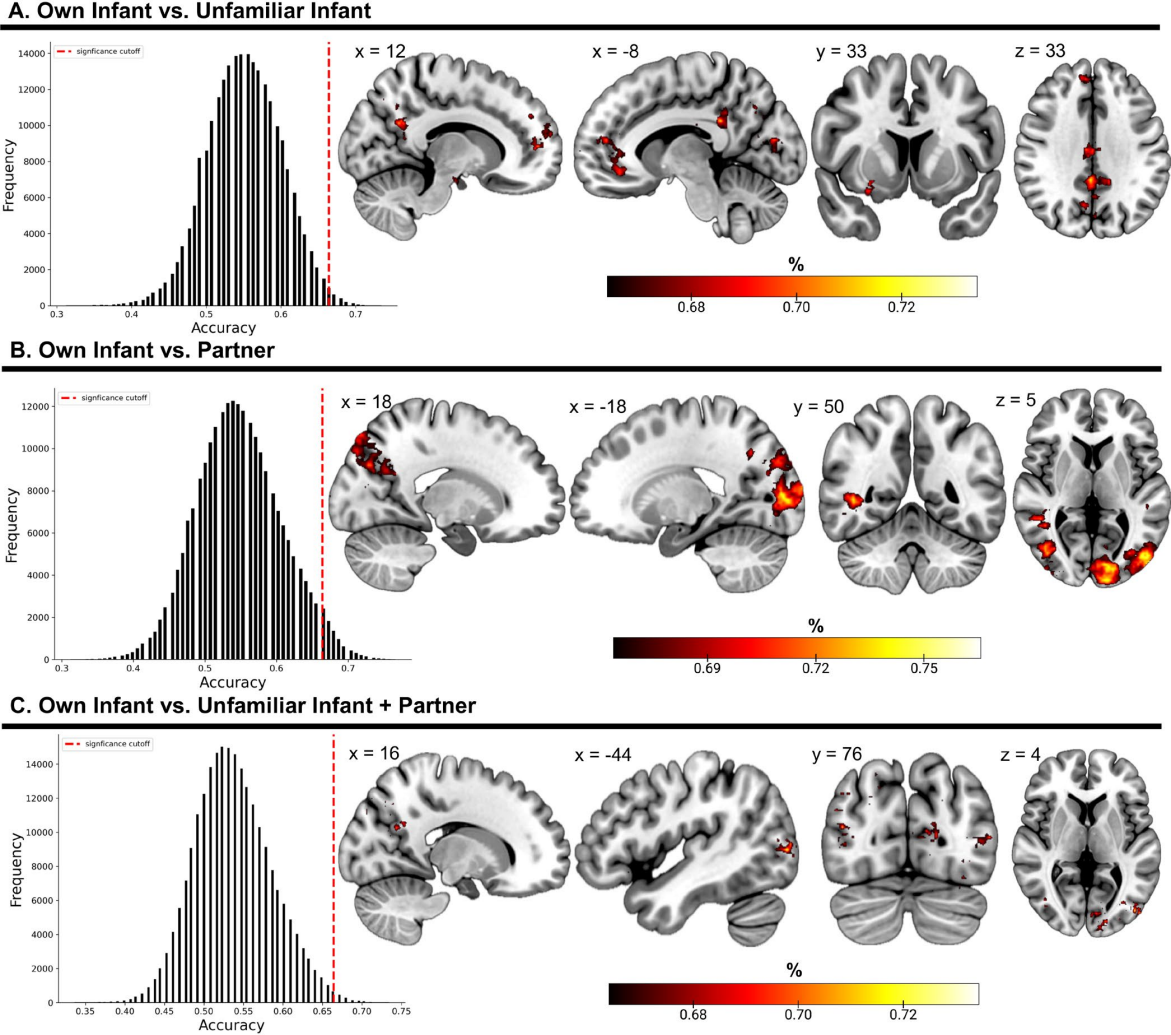
Whole‐brain searchlight analyses determining where in the brain task stimuli are discriminable. Each histogram shows a distribution of accuracy levels (*x*‐axis) across the brain. The y‐axis depicts the number of voxels corresponding to each level of accuracy. The red line indicates our significance cutoff (66.4%), which corresponds to a corrected alpha of 6.89 × 10^−5^. Brain accuracy maps are presented from sagittal (*x*), coronal (*y*), and axial (*z*) perspectives and represent voxels with at least 66.4% classification accuracy. (A) Histogram and accuracy map indicating where in the brain participants distinguish between their own infant and an unfamiliar infant. (B) Histogram and accuracy map showing brain regions classifying participants' own infant and their partner. (C) Histogram and accuracy map of regions distinguishing between activation patterns to own‐infant stimuli versus unfamiliar‐infant and partner stimuli.

A series of whole‐brain searchlight analyses assessed discriminability between task stimuli throughout the brain. An initial searchlight revealed discriminant patterns of activation to each participant's own infant and an unfamiliar infant in the PCC (extending to the precuneus; 73%), OFC (extending to the ACC; 70%), anterior PFC (extending to the dlPFC; 71%), cuneus (70%), IFG (70%), precuneus (70%), parahippocampus (69%), and temporal pole (68%; Figure 4A).

A second searchlight found the visual association area extending through the precuneus and middle occipital gyrus (77%), precuneus (75%), fusiform gyrus extending through the middle temporal gyrus (75%), precentral gyrus (71%), precuneus (71%), middle temporal gyrus (69%), cuneus (69%), and STG (69%) were able to distinguish between participants' own infant and their partner (Figure 4B).

A final searchlight revealed the visual association area (73%) and precuneus (73%) discriminate participants' own infants from the unfamiliar infant and partner stimuli (Figure 4C). Cluster information for each searchlight can be found in the Supplement (Table S3).

### 3.4 Infancy and Familiarity Classification

**FIGURE 5:**
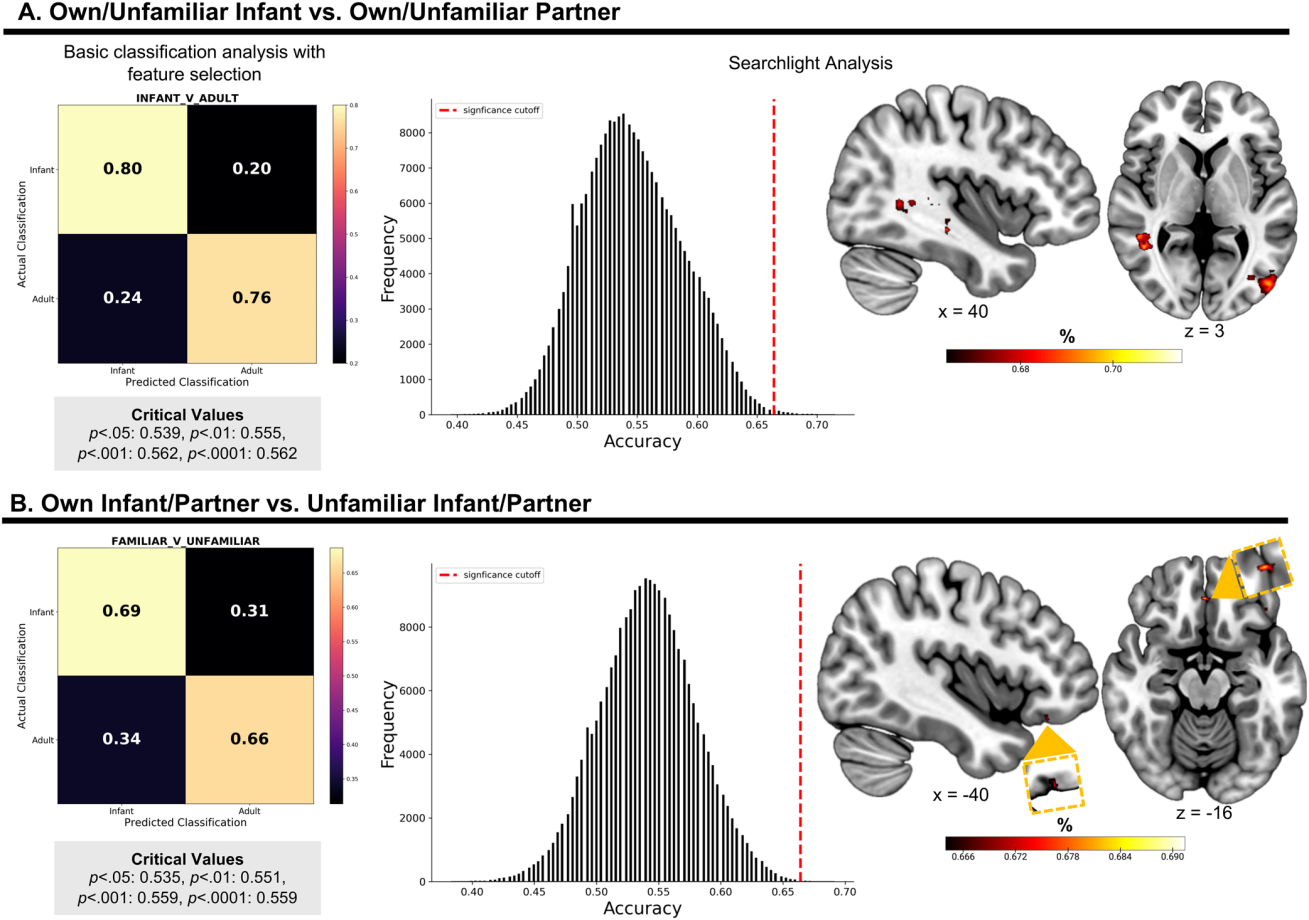
Classifying infancy and familiarity using MVPA with feature selection and searchlight analyses. Confusion matrices show how well the classifier distinguished (A) infant versus adult stimuli and (B) familiar versus unfamiliar stimuli, as well as the percentage of misclassifications. Histograms and brain maps represent results from the (A) infant versus adult and (B) familiar versus unfamiliar searchlight analyses. Critical values for each MVPA with feature selection were determined by a simulated distribution from 1000 permutations, while the significance threshold for the searchlight analyses remained 66.4% as determined by a resel‐wise Bonferroni adjustment.

A basic classification analysis considering only the top 10% of contributing voxels distinguished infant/adult videos with 78.1% accuracy (Figure 5A) and familiar/unfamiliar stimuli with 67.2% accuracy (Figure 5B). Both predictions were significantly above chance (both *p*s < 0.0001) as determined by permutation testing. Follow‐up searchlight analyses revealed the visual association area (72%), middle temporal gyrus (70%), visuomotor area (70%), hippocampus (70%), superior temporal gyrus (70%), parahippocampus (70%), STG (69%), posterior cingulate (68%), and precuneus (70%) classified between infant and adult stimuli with greater than chance accuracy (Figure 5A). Participants' level of familiarity with the stimulus (own vs. familiarity) was distinguishable by a small cluster in the OFC (68%; Figure 5B). Cluster information for these additional analyses can be found in the Supplement (Table S3).

## 4 Discussion

We used both univariate and MVPA approaches to contrast first‐time fathers' neural responses to videos of their own infant, an unfamiliar infant, and their romantic partner. Our findings build on, and extend, the existing literature in several ways. First, we replicated previous work comparing parents' responses to own‐infant and unfamiliar‐infant stimuli, finding stronger activation in mentalizing and emotion regulation regions when fathers view their own baby. We also included an own‐partner condition to introduce another personally relevant and familiar stimulus. The contrast of own‐infant and own‐partner conditions revealed significant activation only in the precuneus. In addition, fathers' activation to their own infants in posterior midline regions was associated with their self‐reported parenting experiences. Specifically, fathers' activation to their own infant versus an unfamiliar infant in the precuneus was positively associated with their prenatal and postpartum bonding with their infant and inversely related to their parenting stress. Fathers' activation in the PCC was also positively associated with their prenatal bonding and negatively associated with their parenting stress. Similarly, fathers' activation in the posterior cingulate and precuneus when viewing their own infants versus their romantic partners was positively associated with their prenatal bonding and negatively associated with postpartum father‐infant bonding problems.

We also leveraged MVPA to better understand which regions were most important for differentiating between own‐infant and unfamiliar‐infant conditions (familiarity versus novelty) and own‐infant and own‐partner responses (infant vs. adult). We found that mentalizing and reward processing regions, as well as the parahippocampus, distinguished between a participant's own infant and an unfamiliar infant, while visual processing regions distinguished own infant versus partner. Exploratory classification analyses revealed infancy (vs. adulthood) was significantly discernible in vision, mentalizing, and emotion processing regions, and stimulus familiarity was distinguishable in the OFC. In sum, our study provides evidence for distinct neural responses to self‐relevant and infant stimuli and suggests a crucial role of mentalizing, reward, and affective regions in fathers' perceptions of their infants.

### 4.1 Univariate Analyses Reveal Heightened Cortical Activation to Own‐Infant Stimuli

As expected, activation in regions involved in mentalizing (PCC, precuneus, dmPFC, angular gyrus, and temporal pole) and emotion regulation (dlPFC, IFG, OFC) was greater when participants viewed videos of their own infant compared to videos of other infants. In addition, we found greater activation in a cluster spanning the precuneus, cuneus, PCC, and middle occipital gyrus for the own‐infant > partner contrast. Our findings are consistent with prior work that has used similar paradigms and add additional insights about differences in paternal neural responses to own‐infant versus romantic partner stimuli. Most of the literature on neural sensitivity to one's own infant has been conducted in mothers and has found activation in cortical and subcortical mentalizing, embodied simulation, and emotion regulation regions (Feldman 2015; Parsons et al. 2017; Paul et al. 2019; Rigo et al. 2019). The limited work on fathers has highlighted cortical networks in response to own‐infant stimuli, such as the PFC, STS, IFG, PCC, OFC, and occipital areas (Provenzi et al. 2021). Posterior midline regions, particularly the precuneus and PCC, are known to be critical for “affective” mentalizing or interpreting others' emotional states (Abu‐Akel and Shamay‐Tsoory 2011; Takahashi et al. 2015). In addition, the precuneus, along with other cortical midline areas, has been shown to undergo structural changes across the transition to parenthood (Hoekzema et al. 2017), and greater decreases in precuneus volume in fathers specifically have been linked to stronger activation when viewing their own infant (Paternina‐Die et al. 2020). Given that infants are unable to communicate their needs verbally, fathers may engage mentalizing networks more strongly during this time to perceive their infants' emotional states (Cárdenas et al. 2021).

Notably, the partner and own‐infant videos were collected at different timepoints (prenatally and postpartum, respectively), which may introduce confounds. Fathers participated in the prenatal interaction with their partners, so they might have some memory of the interaction from which the clips were drawn. The partners were pregnant when the partner videos were recorded, so partner stimuli were also, arguably, infant‐relevant (although the video clips featured only the head and upper torso, meaning that the pregnancy was not a focal point). This may limit our ability to directly compare these stimuli.

### 4.2 Brain Responses to Own‐Infant Stimuli Are Associated With Paternal Bonding and Stress

Brain activation in response to own‐infant cues in the bilateral PCC and precuneus was positively associated with both antenatal and postpartum father–infant bonding and negatively associated with father‐infant bonding problems. Even before birth, fathers' feelings of bonding with their infant in utero predicted their subsequent neural activation to infant stimuli at 6 months postpartum. The connection parents feel towards their infant during pregnancy represents an emerging self‐reported love and sense of protection (Walsh 2010), and cognitive attributions of their infant postpartum may be predicted by this parent‐to‐child feeling of closeness. Further, parenting stress at 3 months postpartum was negatively associated with PCC and precuneus activation in response to the own‐infant–unfamiliar‐infant contrast. Exploratory analyses on the own‐infant–partner contrast revealed that precuneus and posterior cingulate activation were positively associated with prenatal bonding and were inversely associated with bonding problems. Together, our findings suggest that greater posterior mentalizing network processing of own‐infant cues, in contrast with both familiar and unfamiliar stimuli, may indicate more positive adjustment to fatherhood.

### 4.3 Searchlight Analyses Capture Discrete Neural Patterns Across Task Stimuli

Results from our main searchlight analysis reveal discriminability between own‐infant and unfamiliar‐infant stimuli in key mentalizing, visual, and emotion processing regions. Specifically, high classification accuracy was found in the PCC, precuneus, cuneus, aPFC, dlPFC, OFC, IFG, and the temporal pole—cortical areas implicated in general social, emotional, and visual processing (Goodkind et al. 2012; Li et al. 2014; Olson et al. 2007) and paternal responses to own‐infant stimuli (Provenzi et al. 2021). Surprisingly, the parahippocampus was the only subcortical region to discriminate between the two infant‐related stimuli above chance. While the parahippocampus is theorized to be part of a network of regions implicated in processing social emotions (Immordino‐Yang and Singh 2011) and has been reported in some maternal studies on infant processing (Musser et al. 2012; Zhang et al. 2020), we expected to see strong discriminability in subcortical regions more established in the literature on parental own‐infant processing, like the amygdala and striatum. On the one hand, our naturalistic stimuli displayed limited emotion dynamics and may not have been well suited for provoking these more conserved salience detection networks like in previous studies on maternal responses to infant stimuli (Bjertrup et al. 2019). On the other hand, most research on fathers' neural processing of own‐infant visual stimuli, including our studies, has not detected significant activation differences in subcortical networks. Interestingly, recent work from our group on structural changes across the transition to fatherhood revealed significant cortical, but not subcortical, shrinkage from prenatal to postpartum timepoints in fathers, in contrast with prior work on mothers that found both cortical and subcortical change (Martínez‐García et al. 2023; Saxbe and Martínez‐García 2024).

Discriminability between own‐infant and partner stimuli was observed in mentalizing and visual processing regions, including the visual association area, precuneus, middle occipital gyrus, fusiform gyrus, and middle temporal gyrus, emphasizing the roles of self‐referential processing and visual discrimination in distinguishing self‐relevant stimuli. These findings are consistent with previous research showing the involvement of visual and cognitive areas in processing familiar faces and emotionally relevant stimuli (Rutherford et al. 2015). Finally, as hypothesized, the precuneus and visual association areas differentiated father's own infant from our unfamiliar‐infant + partner condition, highlighting the importance of these regions in distinguishing self‐relevant infant stimuli from familiar and unfamiliar adults (Herold et al. 2016; Lombardo et al. 2010). Overall, MVPA provided additional evidence for distinct neural patterns in mentalizing, visual, and affective regions to viewing your own infant in first‐time fathers.

### 4.4 Familiarity and Infant Schema Contribute to Neural Discriminability

In our exploratory analyses, we used MVPA approaches to explore the general neural discernment of infancy and familiarity. Binary classification selecting for the top 10% of informative voxels revealed above‐chance discrimination of stimulus infancy and familiarity. The searchlights found self‐relevance (own‐infant + partner vs. unfamiliar‐infant + unfamiliar‐partner) to be classified in a small OFC cluster which aligns with previous MVPA studies that suggest higher‐order mentalizing regions play a role in discriminating between familiar and unfamiliar people (Kruse et al. 2016; Thornton and Mitchell 2017; Visconti di Oleggio Castello et al. 2017). We were unable to measure the extent to which the faces of the infants (either own or unfamiliar infant) resembled the faces of the fathers participating in the study. It is possible that self‐resemblance is one aspect of own‐infant face processing that gives infants a unique salience. That is, infants often look like their fathers, so the fathers recognize their features in their own infants' faces. It is also possible that the unfamiliar infants bore a resemblance to some of the participating fathers, which may have added unexplained variance. The question of self‐resemblance is an important one that warrants further study.

Infant and adult stimuli were also significantly classified by the top 10% of contributing voxels (~78%). Our searchlight analysis found significant clusters in the visual association area, middle temporal gyrus, parahippocampus, STG, precuneus, and the PCC, each peaking between 69%–72% accuracy. As a group, these support higher‐order visual processing, emotional processing of social information, social cognition, and semantic association of sensory patterns (Immordino‐Yang and Singh 2013; Kellenbach et al. 2005; Visconti di Oleggio Castello et al. 2021). Infant faces generally have highly distinct morphological features compared to adults, which likely facilitates highly accurate discrimination. Furthermore, evolutionary theory suggests that these features prime salient responses and promote quick attentional shifts towards infants—especially infants displaying emotion (Brosch et al. 2008; Lorenz 1981; Thompson‐Booth et al. 2014). Regions such as the superior temporal gyrus and the precuneus may be involved in the identification of these visual patterns as containing salient and potentially goal‐relevant information (Glocker et al. 2009; Plank et al. 2022).

### 4.5 Strengths and Limitations

To our knowledge, this study is the first fMRI study comparing parents' neural activation to both their own infant and their own partner. Thus, our findings provide greater understanding of unique and overlapping neural representations of familial stimuli. Second, this study used a racially and ethnically diverse sample, which increases external validity. Third, this study employed a traditional univariate approach to replicate and extend previous studies on own‐infant processing and complemented these analyses with MVPA to capture differences in spatially distributed activation patterns across the cortex and subcortex. Together, these approaches provide converging evidence for the distinctiveness of own‐infant neural responses relative to other socially salient conditions.

Despite its many strengths, this study has several limitations. First, although our sample of 32 fathers is larger than many parental brain studies, it is still relatively modest. While we used leave‐one‐out cross‐validation to validate our models out‐of‐sample classification accuracy, we encourage future studies with larger samples to replicate. Second, while our sample is ethnically diverse, it is also highly educated and mostly limited to families from Southern California, USA. Future work should consider including a wider array of sociodemographic groups, fathers in same‐sex couples, as well as mothers and non‐biological parents. Relatedly, unfamiliar‐person conditions were standardized, not demographically matched, across participants. While our approach controls for feature variability across unfamiliar stimuli, this may introduce noise into our familiarity‐based analyses. Third, parents' neural responses to their romantic partner may be modulated by the strength of their bond (Numan and Young 2016). While beyond the scope of the current project, future research can examine whether couple relationship quality is associated with partner neural processing in comparison to infant stimuli. The current manuscript did not test the partner > unfamiliar woman contrast since the current study focuses on own‐infant stimuli, but we plan to further explore the partner stimuli in future work. A related limitation is that the videos of fathers' partners were collected when the partner was pregnant, which may have affected how the father processes their partner's image. Fourth, we do not have a control group of non‐fathers and thus cannot definitively conclude that our results are unique to fathers. However, other studies on own‐infant neural processing have also focused on fathers without a comparison group because the “own child” condition can only be tested with fathers (Açil et al. 2025). Fifth, we computed group‐level, instead of subject‐level, MVPA maps to help reduce variance in our training data, which is an important consideration when employing machine‐learning approaches in smaller datasets. In consequence, we were unable to test associations between subject‐level searchlight maps and individual difference measures, which limits comparison between our two analytic approaches. Lastly, a wider range of infant‐related stimuli and degree of familial relevance (family, friends, strangers that have been seen before, unknown strangers, etc.) would allow for representational similarity analyses of neural patterns that can further disentangle the dimensional contributions of various stimuli aspects.

## 5 Conclusion

This study supports the existing literature on parents' processing of own‐infant stimuli and extends it by introducing a novel contrast (own‐partner) and novel methods (MVPA). Our findings suggest that neural processing of own‐infant stimuli is distinct from other familiar (own‐partner) and infant (unfamiliar‐infant) stimuli. Like in prior studies, a univariate approach revealed neural sensitivity to own‐infant stimuli in cortical areas subserving mentalizing processes, emotion regulation, and reward processing. Activation in the posterior midline of the brain (precuneus and PCC) to own‐infant stimuli was associated with father–infant bonding and postpartum parenting stress, indicating a relationship between neural processing of one's own child and adjustment to fatherhood. Searchlight analyses revealed unique patterns of activation in similar regions to own‐infant processing, with the addition of the parahippocampus and various visual processing regions. Finally, exploratory MVPA revealed distinct activation patterns when differentiating infant versus adult and familiar versus unfamiliar stimuli, indicating these features may contribute to fathers' unique neural processing of their own baby. Further research is needed to replicate these findings and continue to explore how fathers respond distinctly to their own infants and how these responses map onto their parenting experiences and behaviors.

## Conflicts of Interest

The authors declare no conflicts of interest.

## Supporting information


**Data S1:** Supporting Information.

## Data Availability

The data that support the findings of this study are available from the corresponding author upon reasonable request.
