# Supplementary material for: My Baby Versus the World: Fathers' Neural Processing of Own‐Infant, Unfamiliar‐Infant, and Romantic Partner Stimuli
Source: Hum Brain Mapp. 2025 Aug 12;46(11):e70324. doi: 10.1002/hbm.70324 (PMC12340546; doi:10.1002/hbm.70324)
Supplement: Supplementary file 1 — Data S1: Supporting Information. [file HBM-46-e70324-s001.docx]

**My baby versus the world: fathers’ neural processing of own-infant, unfamiliar-infant, and romantic partner stimuli**

(*Supplementary Materials*)

**Table S1**

*Whole-brain cluster information from univariate analyses*

|  | **MNI Coordinates** | | | **Voxels** | **Z_MAX_** |
| --- | --- | --- | --- | --- | --- |
|  | **X** | **Y** | **Z** |  |  |
| ***Own Infant > Unfamiliar Infant*** |  |  |  |  |  |
| Precuneus, Posterior Cingulate | -2 (7) | -64(-60) | 16(26) | 5130 | 5.19 |
| Dorsomedial Anterior Prefrontal Cortex | 0(-2) | 52(50) | 22(17) | 2963 | 5.42 |
| Angular Gyrus | -56(-49) | -60(-62) | 20(19) | 674 | 4.54 |
| Inferior Frontal Gyrus, Dorsolateral Prefrontal Cortex | 48(45) | 20(21) | 24(22) | 549 | 4.55 |
| Orbitofrontal Cortex | -42(-38) | 20(21) | -16(-15) | 460 | 4.64 |
| Orbitofrontal Cortex | 28(29) | 16(18) | -20(-21) | 177 | 4.54 |
| Temporal Pole | -52(-48) | 4(1) | -30(-28) | 154 | 4.00 |
| ***Own Infant > Partner*** |  |  |  |  |  |
| Precuneus | 18(5) | -64(-72) | 28(33) | 7846 | 6.12 |

Note: Values in parentheses represent centre of gravity (COG) values.

**Table S2**

*Cluster information from permutation-based analyses*

|  | **MNI Coordinates** | | | **Voxels** | **1-*p*** | ***r*_MAX_** |
| --- | --- | --- | --- | --- | --- | --- |
|  | **X** | **Y** | **Z** |  |  |  |
| ***Own Infant > Unfamiliar Infant*** |  |  |  |  |  |  |
| **AEA** |  |  |  |  |  |  |
| Posterior Cingulate, Precuneus | -10(-4) | -60(-68) | 14(20) | 237 | 0.99 | 0.66(0.50) |
| **PPNAS** |  |  |  |  |  |  |
| Precuneus | 6(6) | -72(70) | 34(34) | 10 | 0.96 | 0.57(0.56) |
| **PSI** |  |  |  |  |  |  |
| Precuneus, Posterior Cingulate | 0(-2) | -68(-66) | 26(24) | 307 | 0.98 | -0.59(-0.50) |
| **PBQ** |  |  |  |  |  |  |
| Precuneus, Posterior Cingulate | -2(-1) | -66(-63) | 34(24) | 454 | 0.99 | -0.64(-0.51) |
| ***Own Infant > Partner*** |  |  |  |  |  |  |
| **AEA** |  |  |  |  |  |  |
| Posterior Cingulate, Precuneus | -2(-6) | -64(-70) | 14(20) | 223 | 0.98 | 0.59(0.50) |
| Precuneus | 4(6) | -74(-74) | 48(50) | 38 | 0.96 | 0.56(0.51) |
| Precuneus | -4(-4) | -62(-68) | 58(54) | 33 | 0.95 | 0.51(0.49) |
| Precuneus | 8(6) | -68(-68) | 34(34) | 28 | 0.96 | 0.56(0.51) |
| **PBQ** |  |  |  |  |  |  |
| Precuneus | -4(8) | -60(-73) | 8(32) | 2058 | >0.99 | -0.73(-0.47) |

Note: MNI coordinates and 1-*p* values reflect cluster peak information. A 1-*p* value > 0.95 corresponds to *p*<.05. Clusters outputted from FSL’s *cluster* tool that were <10 voxels are not included in the table. Values in parentheses represent centre of gravity (COG) or mean values.

**Table S3**

*Cluster information from whole-brain searchlight analyses*

|  | **MNI Coordinates** | | | **Voxels** | **Accuracy_MAX_** |
| --- | --- | --- | --- | --- | --- |
|  | **X** | **Y** | **Z** |  |  |
| ***Own Infant vs. Unfamiliar Infant*** |  |  |  |  |  |
| Posterior Cingulate, Precuneus | 2(2) | -42(-52) | 32(30) | 733 | 0.73 |
| Orbitofrontal Cortex, Anterior Cingulate | -6 (-4) | 34(40) | -12(2) | 327 | 0.70 |
| Anterior Prefrontal Cortex, Dorsolateral Prefrontal Cortex | 12(10) | 52(52) | 8(22) | 228 | 0.71 |
| Cuneus | -12(-10) | -86(-78) | 12(12) | 198 | 0.70 |
| Orbitofrontal Cortex | -38(-38) | 28(30) | -22(-12) | 171 | 0.71 |
| Posterior Cingulate | 6(4) | -16(-18) | 32(34) | 160 | 0.71 |
| Posterior Cingulate | 4(4) | -54(-58) | 8(8) | 76 | 0.71 |
| Inferior Frontal Gyrus | 52(54) | 20(20) | 6(5) | 51 | 0.70 |
| Orbitofrontal Cortex | 28(26) | 8(10) | -16(-14) | 34 | 0.70 |
| Precuneus | 18(20) | -66(-62) | 44(40) | 29 | 0.70 |
| Parahippocampus | 10(10) | -10(-12) | -20(-16) | 23 | 0.69 |
| Temporal Pole | -38(-38) | 18(18) | -38(-36) | 19 | 0.68 |
| Temporal Pole | 24(26) | 20(20) | -32(-30) | 16 | 0.67 |
| Precuneus | 12(12) | -58(-58) | 38(40) | 14 | 0.68 |
| Posterior Cingulate | -8(-8) | -22(-22) | 26(24) | 10 | 0.68 |
| Anterior Prefrontal Cortex | -4(-4) | 58(56) | 2(4) | 10 | 0.68 |
| ***Own Infant vs. Partner*** |  |  |  |  |  |
| Visual Association Area, Middle Occipital Gyrus | -44(-24) | -76(-82) | 6(10) | 4155 | 0.77 |
| Precuneus | 24(28) | -66(-76) | 28(26) | 2843 | 0.75 |
| Fusiform Gyrus | 42(46) | -50(-34) | 2(-2) | 679 | 0.75 |
| Fusiform Gyrus, Middle Temporal Gyrus | -42(-48) | -32(-34) | -12(-4) | 139 | 0.70 |
| Precentral Gyrus | 52(50) | -4(-2) | 44(48) | 125 | 0.71 |
| Visual Association Area | 34(32) | -80(-70) | -20(-16) | 82 | 0.70 |
| Precuneus | -18(-18) | -64(-64) | 38(36) | 25 | 0.71 |
| Visual Association Area | 20(22) | -82(-80) | -18(-18) | 20 | 0.68 |
| Middle Temporal Gyrus | 64(64) | -8(-6) | -22(-22) | 19 | 0.69 |
| Cuneus | 8(10) | -84(-84) | 14(14) | 15 | 0.69 |
| Visual Association Area | -40(-40) | -85(-85) | -12(-12) | 11 | 0.69 |
| Superior Temporal Gyrus | 70(70) | -28(-26) | 4(4) | 10 | 0.69 |
| Precuneus | -28(-28) | -60(-62) | 36(38) | 10 | 0.68 |
| ***Own Infant vs. Unfamiliar Infant + Partner*** |  |  |  |  |  |
| Visual Association Area | -44(-22) | -80(-84) | 4(6) | 645 | 0.73 |
| Precuneus | 22(24) | -80(-72) | 30(26) | 476 | 0.73 |
| Visual Association Area | -28(-28) | -70(-70) | -20(-20) | 31 | 0.70 |
| Visual Association Area | 44(40) | -80(-80) | 26(26) | 25 | 0.70 |
| Visual Association Area | 40(42) | -76(-74) | 34(32) | 14 | 0.70 |
| Precuneus | 16(18) | -62(-66) | 42(42) | 12 | 0.70 |
| Visual Association Area | -32(-36) | -76(-78) | -16(-16) | 11 | 0.69 |
| Visual Association Area | -54(-8) | -70(-70) | -8(-8) | 10 | 0.69 |
| ***Infant vs. Adult*** |  |  |  |  |  |
| Visual Association Area | -46(-46) | -76(-76) | 4(4) | 182 | 0.72 |
| Middle Temporal Gyrus | 44 (44) | -50(-48) | 4(2) | 126 | 0.70 |
| Parahippocampus | 40(40) | -24(-24) | -12(-8) | 26 | 0.70 |
| Superior Temporal Gyrus | 52(54) | -12(-12) | -8(-6) | 17 | 0.69 |
| Superior Temporal Gyrus | 44(44) | -34(-34) | 6(6) | 14 | 0.68 |
| Posterior Cingulate | -28(-30) | -72(-74) | 16(16) | 12 | 0.68 |
| Precuneus | 20(18) | -80(-80) | 54(54) | 12 | 0.70 |
| ***Familiar vs. Unfamiliar*** |  |  |  |  |  |
| Orbitofrontal Cortex | -40(-40) | 32(32) | -18(-16) | 10 | 0.68 |

Note: Clusters outputted from FSL’s *cluster* tool that were <10 voxels are not included in the table. Values in parentheses represent centre of gravity (COG) values.
